# Automated Low-Cost Smartphone-Based Lateral Flow Saliva Test Reader for Drugs-of-Abuse Detection

**DOI:** 10.3390/s151129569

**Published:** 2015-11-24

**Authors:** Adrian Carrio, Carlos Sampedro, Jose Luis Sanchez-Lopez, Miguel Pimienta, Pascual Campoy

**Affiliations:** 1 Computer Vision Group, Centre for Automation and Robotics (UPM-CSIC), Calle José Gutiérrez Abascal 2, Madrid 28006, Spain; E-Mails: carlos.sampedro@upm.es (C.S.); jl.sanchez@upm.es (J.L.S.-L.); pascual.campoy@upm.es (P.C.); 2 Aplitest Health Solutions, Paseo de la Castellana 164, Madrid 28046, Spain; E-Mail: mp@aplitest.com

**Keywords:** smartphone, drugs-of-abuse, diagnostics, computer vision, machine learning, neural networks

## Abstract

Lateral flow assay tests are nowadays becoming powerful, low-cost diagnostic tools. Obtaining a result is usually subject to visual interpretation of colored areas on the test by a human operator, introducing subjectivity and the possibility of errors in the extraction of the results. While automated test readers providing a result-consistent solution are widely available, they usually lack portability. In this paper, we present a smartphone-based automated reader for drug-of-abuse lateral flow assay tests, consisting of an inexpensive light box and a smartphone device. Test images captured with the smartphone camera are processed in the device using computer vision and machine learning techniques to perform automatic extraction of the results. A deep validation of the system has been carried out showing the high accuracy of the system. The proposed approach, applicable to any line-based or color-based lateral flow test in the market, effectively reduces the manufacturing costs of the reader and makes it portable and massively available while providing accurate, reliable results.

## 1. Introduction

Most rapid tests or qualitative screening tests on the market are chromatographic immunoassays. They are used to detect the presence or absence of a substance (analyte) in an organic sample. The result is obtained in a few minutes and without the need of specialized processes or equipment. The use of this kind of test is an aid in the rapid diagnose of different diseases (*i.e.*, HIV, hepatitis, malaria, *etc.*) or certain physiological conditions (pregnancy, drugs-of-abuse, blood glucose levels, cholesterol, *etc.*).

In the particular case of drug-of-abuse detection, tests are commonly based on the principle of competitive binding: drugs that may be present in the organic sample (*i.e.*, urine, saliva, *etc.*) compete against a drug conjugate, present in the test strip, for the specific binding sites of the antibody. During the test procedure, the sample migrates along the test strip by capillary action.

If a substance present in the sample is available in a concentration lower than the cutoff level, it will not saturate the binding sites of the particles coated with the antibody on the test strips. The coated particles will be then captured by the immobilized conjugate of each substance (drug), and a specific area in the strip will be visibly colored. No coloration will appear in this area if the concentration of the substance is above the cutoff level, as it will saturate all of the binding points of the specific antigen for such a substance. An additional control area is usually disposed in the test strip and colored upon effective migration through the initial test area, to confirm the validity of the test result.

**Figure 1:**
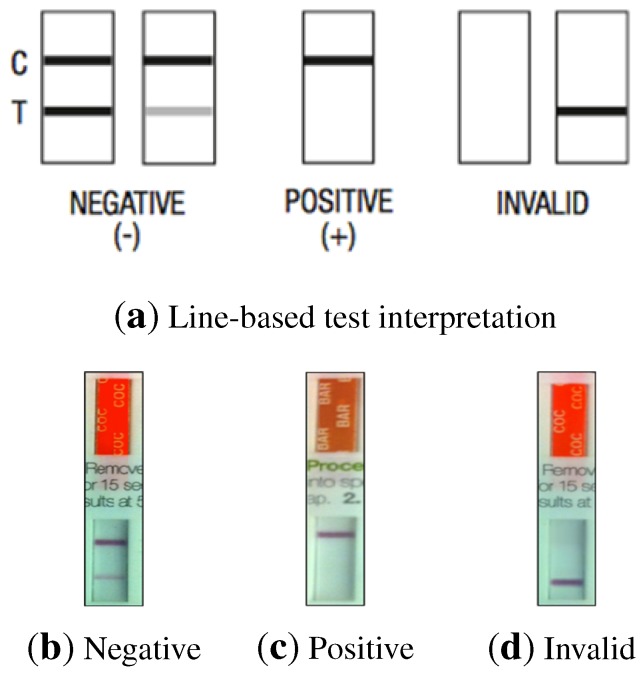
(**a**) Line-based test interpretation samples; (**b**–**d**) Strip samples.

**Figure 2:**
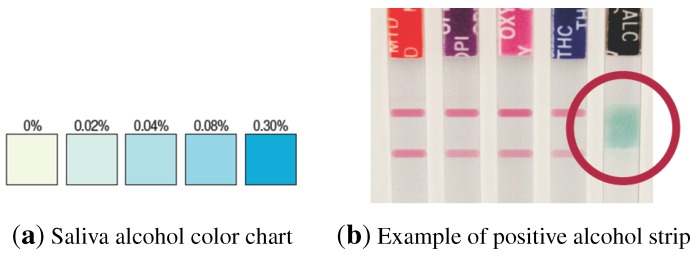
(**a**) Color chart to obtain the relative blood alcohol concentration by comparison with the colored area in the test strip; (**b**) test sample including an alcohol strip.

Most of the results obtained using the commercially-available test kits are interpreted visually by a human operator, either by the presence or absence of colored lines (Figure 1) or by comparison of the changes in color of particular areas of a test strip against a pattern (Figure 2). Some of the problems arising from the use of rapid/screening tests are the following:

- Interpretation: This is done by direct visual inspection, and thus, the results interpretation may vary depending on the human operator (training, skills, lighting conditions, *etc.*). Normally, under well-lit environments, there are less interpretation errors than when this kind of test is used under poor light conditions, as this may affect the ability of the operator to judge the result correctly (*i.e.*, testing for drugs during a roadside control in the middle of the night).
- Confirmation is required: Any results obtained should be confirmed using a technique with a higher specificity, such as mass spectrometry, specially when presumptive positive results are obtained.
- Conditions under which the tests are performed: Rapid tests are used in conditions where there is no availability of specialized equipment.
- Dispersion and structure of the data: Test results and subject data are scattered and stored, usually in paper-based format.
- Processing and analysis of data: Data for decision-making are gathered and analyzed manually by human operators, being prone to human errors.

While most lateral flow tests are intended to operate on a purely qualitative basis, it is possible to measure the color intensity of the test lines in order to determine the quantity of analyte in the sample. Computer vision has been proven as a useful tool for this purpose, as capturing and processing test images can provide objective color intensity measurements of the test lines with high repeatability. By using a computer vision algorithm, the user-specific bias is eliminated (ability to interpret), which may affect the result obtained.

Smartphones’ versatility (connectivity, high resolution image sensors and high processing capabilities, use of multimedia contents, *etc.*) and performance condensed in small and lightweight devices, together with the current status of wireless telecommunication technologies, exhibit a promising potential for these devices to be utilized for lateral flow tests interpretation, even in the least developed parts of the world.

In this paper, we present a novel algorithm to qualitatively analyze lateral flow tests using computer vision and machine learning techniques running on a smartphone device. The smartphone and the tests are contained on a simple hardware system consisting of an inexpensive 3D-printed light box.

The light box provides controlled illumination of the test during the image capture process while the smartphone device captures and processes the image in order to obtain the result. Test data can be then easily stored and treated in a remote database by taking advantage of the smartphone connectivity capabilities, which can help to increase the efficiency in massive drug-of-abuse testing, for example in roadside controls, prisons or hospitals.

This approach effectively reduces the manufacturing costs of the reader, making it more accessible to the final customer, while providing accurate, reliable results. To the authors’ knowledge, the interpretation of lateral flow saliva tests for drug-of-abuse detection using computer vision and machine learning techniques on a smartphone device is completely novel.

The remainder of the paper is organized as follows. Firstly, a review of the state of the art for hand-held diagnostic devices, in general, and for drug-of-abuse lateral flow readers, in particular, is presented. Secondly, the saliva test in which the solution was implemented is introduced. Thirdly, the software and hardware solutions proposed are described. Fourthly, the methodology for validating the results is discussed. Then, the results of the evaluation through agreement and precision tests are shown. Finally, conclusions are presented.

## 2. State of the Art

Martinez *et al.* [1] used paper-based microfluidic devices for running multiple assays simultaneously in order to clinically quantify relevant concentrations of glucose and protein in artificial urine. The intensity of color associated with each colorimetric assay was digitized using camera phones. The same phone was used to establish a communications infrastructure for transferring the digital information from the assay site to an off-site laboratory for analysis by a trained medical professional.

A lens-free cellphone microscope was developed by Tseng *et al.* [2] as a mobile approach to provide infectious disease diagnosis from bodily fluids, as well as rapid screening of the quality of water resources by imaging variously-sized micro-particles, including red blood cells, white blood cells, platelets and a water-borne parasite (*Giardia lamblia*). Improved works in this field were published by Zhu *et al.* [3], who also developed a smartphone-based system for the detection of *Escherichia coli* [4] on liquid samples in a glass capillary array.

Matthews *et al.* [5] developed a dengue paper test that could be imaged and processed by a smartphone. The test created a color on the paper, and a single image was captured of the test result and processed by the phone, quantifying the color levels by comparing them with reference colors.

Dell *et al.* [6] presented a mobile application that was able to automatically quantify immunoassay test data on a smartphone. Their system measured both the final intensity of the capture spot and the progress of the test over time, allowing more discriminating measurements to be made, while showing great speed and accuracy. However, registration marks and an intensity calibration pattern had to be included in the test to correctly process the image, and also, the use of an additional lens was required for image magnification.

Uses of rapid diagnostic test reader platforms for malaria, tuberculosis and HIV have been reported by Mudanyali *et al.* [7,8]. Smartphone technology was also used to develop a quantitative rapid diagnostic test for multi-bacillary leprosy, which provided quantifiable and consistent data to assist in the diagnosis of MBleprosy.

A smartphone-based colorimetric detection system was developed by Shen *et al.* [9], together with a calibration technique to compensate for measurement errors due to variability in ambient light. Oncescu *et al.* [10] proposed a similar system for the detection of biomarkers in sweat and saliva. Similar colorimetric methods for automatic extraction of the result in ELISA plates [11] and proteinuria in urine [12] have also been reported. However, none of those works presented developments on colored line detection.

In the area of drug-of-abuse detection, accurate confirmatory results are nowadays obtained in a laboratory usually by means of mass spectrometry techniques. These laboratory-based solutions are expensive and time consuming, as the organic sample has to be present in the laboratory in order to perform the analysis. In contrast, screening techniques provide *in situ*, low-cost, rapid presumptive results with a relatively low error rate, with immunoassay lateral flow tests currently being the most common technique for this type of test. Nonetheless, as most lateral flow tests operate on a purely qualitative basis, obtaining a result is usually subject to the visual interpretation of colored areas on the test by a human operator, therefore introducing subjectivity and the possibility of human errors to the test result. Hand-held diagnostic devices, known as lateral flow assay readers, are widely used to provide automated extraction of the test result.

VeriCheck [13] is an example of an automated reader for lateral flow saliva tests consisting of the use of a conventional image scanner and a laptop. However, this solution is far from portable, as it consists of multiple devices and requires at least a power outlet for the scanner.

DrugRead [14] offers a portable automated reader solution on a hand-held device. However, our system offers a low-cost, massively available solution, as it has been implemented on a common smartphone device.

## 3. Saliva Test Description

The proposed image processing methodology can be applied to any line presence or absence-based or color interpretation-based immunoassay tests on the market. For the results and validation presented, the rapid oral fluid drug test DrugCheck SalivaScan [15], manufactured by Express Diagnostics Inc. (Minneapolis, MN, USA), was used. DrugCheck SalivaScan is an immunoassay for rapid qualitative and presumptive detection of drugs-of-abuse in human oral fluid samples.

**Figure 3:**
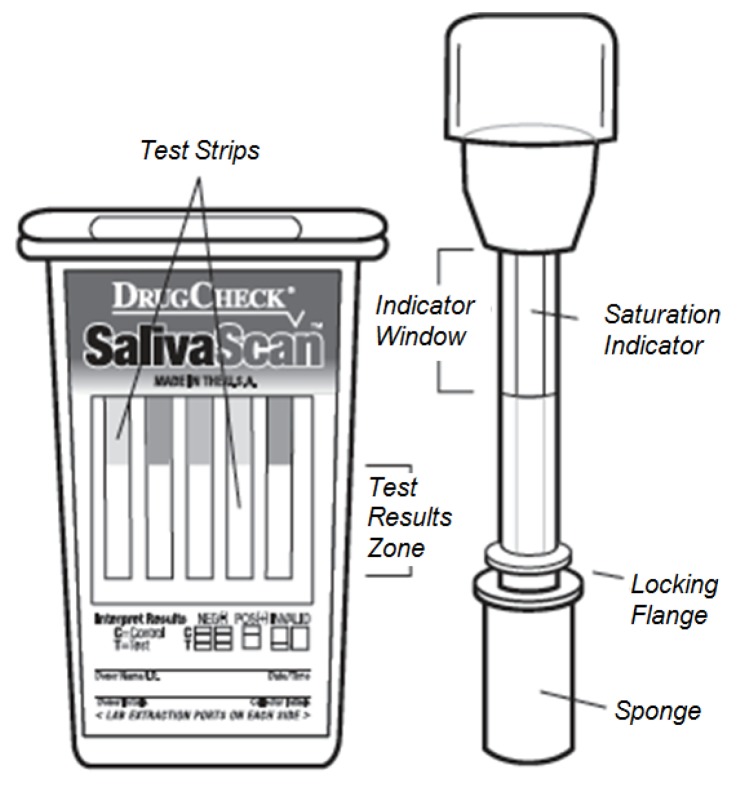
DrugCheck SalivaScan test. During the test procedure, the collection swab (**right**) will be inserted into the screening device (**left**).

The device is made of one or several strips of membrane incorporated in a plastic holder, as shown in Figure 3. For sample collection, a swab with a sponge containing an inert substance that reduces saliva viscosity is used. A saturation indicator is placed inside the collection swab to control the volume of saliva collected and to provide the indication to start the reaction (incubation) of the rapid test. The test may contain any combination of the parameters/substances and cutoff levels as listed in Table 1.

**Table 1:** Calibrators and cutoff levels for different line-based drug tests.

Test	Calibrator	Cutoff Level (ng/mL)
Amphetamine (AMP)	D-Amphetamine	50
Benzodiazepine (BZO)	Oxazepam	10
Buprenorphine (BUP)	Buprenorphine	5
Cocaine (COC)	Benzoylecgonine	20
Cotinine (COT)	Cotinine	50
EDDP (EDDP)	2-Ethylidene-1,5-dimethyl-3,3-diphenylpyrrolidine	20
Ketamine (KET)	Ketamine	50
Marijuana (THC)	11-nor-Δ9-THC-9 COOH	12
Marijuana (THC)	Δ9-THC	50
Methadone (MTD)	Methadone	30
Methamphetamine (MET)	D-Methamphetamine	50
Opiates (OPI)	Opiates	40
Oxycodone (OXY)	Oxycodone	20
Phencyclidine (PCP)	Phencyclidine	10
Propoxyphene (PPX)	Propoxyphene	50
Barbiturate (BAR)	Barbiturate	50

Additionally, the test may include a strip for the detection of the presence of alcohol (ethanol) in oral fluid, providing an approximation of the relative blood alcohol concentration. When in contact with solutions of alcohol, the reactive pad in the strip will rapidly turn colors depending on the concentration of alcohol present. The pad employs a solid-phase chemistry that uses a highly specific enzyme reaction. The detection levels of relative blood alcohol concentration range between 0.02% up to 0.30%.

### 3.1. Test Procedure

The first step in the test procedure consists of saturating the saliva test sponge. For this, the donor sweeps the inside of the mouth (cheek, gums, tongue) several times using a collection swab and holds it in his or her closed mouth until the color on the saturation indicator strip appears in the indicator window.

The collection swab can then be removed from the mouth and inserted into the screening device. Once the specimen is dispersed among all strips, the device should be set and kept upright on a flat surface while the test is running. After use, the device can be disposed of or sent to a laboratory for confirmation on a presumptive positive result.

### 3.2. Interpretation of Results

In the case of non-alcohol strips, interpretation is based on the presence or absence of lines. Two differently-colored lines may appear in each test strip, a control line (C) and a test line (T), leading to different test results, as shown in Figure 1. The areas where these lines may appear are called the control region and the test region, respectively.

Negative results can be read as soon as both lines appear on any test strip, which usually happens within 2 min. Presumptive positive results can be read after 10 min. Three possible results may be obtained:

1. Positive: Only one colored line appears in the control region. No colored line is formed in the test region for a particular substance. A positive result indicates that the concentration of the analyte in the sample exceeds the cutoff level.
2. Negative: Two colored lines appear on the membrane. One line is formed in the control region and another line in the test region for the corresponding substance. A negative result indicates that the analyte concentration is below the cutoff level.
3. Invalid result: No control line is formed. The result of any test in which there is no control line during the specified time should not be considered.

The intensity of the colored line in the test region may vary depending on the concentration of the analyte present in the specimen. Therefore, any shade of color in the test region should be considered negative.

In the case of saliva alcohol strips, the interpretation of the results should be made by comparing the color obtained in the reagent strip against a printed color pattern that is provided with the test (Figure 2). Alcohol strips must be read at 2 min, as pad color may change, and again, three possible results may be obtained:

1. Positive: The test will produce a color change in the presence of alcohol in the sample. The color intensity will range, being light blue at a 0.02% relative blood alcohol concentration and dark blue near a 0.30% relative blood alcohol concentration. An approximation of the relative blood alcohol concentration within this range can be obtained by comparison with the provided color pattern (Figure 2).
2. Negative: If the test presents no color changes, this should be interpreted as a negative result, indicating that alcohol has not been detected in the sample.
3. Invalid: If the color pad is already colored in blue before applying the saliva sample, the test should not be used.

## 4. System Description

In the following section, a description of the saliva test reader system is presented. Firstly, image acquisition aspects are discussed, including a description of the light box device used for illumination normalization purposes. Secondly, the computer vision algorithms used in the image processing stage are described. Finally, the machine learning algorithms used for lateral histogram classification are presented.

### 4.1. Image Acquisition

Image acquisition is an extremely important step in computer vision applications, as the quality of the acquired image will condition all further image processing steps. Images must meet certain requirements in terms of image quality (blur, contrast, illumination, *etc.*). The positions of the camera (a mobile device in our case) and the object to be captured (here, the test) should remain in a constant relative position for the best results. However, contrary to traditional image processing applications, a mobile device is hand-held and, therefore, does not have a fixed position with respect to the test, which can lead to motion blur artifacts. Furthermore, mobile devices are used in dynamic environments, implying that ambient illumination has to be considered in order to obtain repeatable results regardless of the illumination conditions.

**Figure 4:**
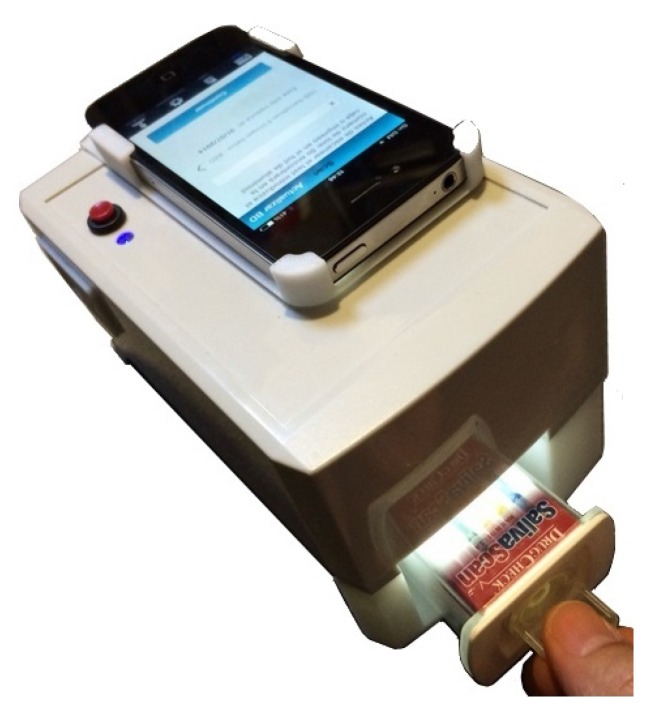
Light box with embedded electronic lighting system, which minimizes the relative movement between the smartphone and the test and the effects of external illumination changes.

In order to minimize all image acquisition-related problems, a small light box was designed to keep the relative position between the smartphone and the test approximately constant, while removing external illumination and projecting white light onto the test with an embedded electronic lighting system. The light box, with dimensions of 150 × 70 × 70 mm, is shown in Figure 4. It is very portable, weighting only 300 g, and it can manufactured at a low cost with a 3D printer.

In order to acquire an image, the saliva test is inserted into the light box; the lighting system is activated using a mounted button, and the smartphone is attached to the light box. The smartphone application has an implemented timer, which allows one to measure the elapsed time and to provide the user with a result as soon as it is available. The test reader provides a result by using a single captured image.

Three smartphone devices were selected for capturing and processing the test images, taking into account their technical specifications and the mobile phone market share: Apple iPhone 4, 4S and 5.

For the purpose of implementing and testing the computer vision and machine learning algorithms, a total of 683 images, containing a total of 2696 test strips, were acquired with the mentioned iPhone models.

### 4.2. Image Preprocessing and Strip Segmentation

Even with an elaborated approach for the image capture procedure, further image processing stages have to deal with image noise and small displacements and rotations of the test within the image, which can be caused by many factors. Just to highlight a few, smartphones might have a loose fit in the light box fastening system; the in-built smartphone cameras come in a variety of resolutions and lenses; furthermore, there might be slight differences in the brightness of the saliva strip’s material.

Once the image has been acquired, the first step is to localize in the image the region corresponding to the strips. For this purpose, this area is manually defined in a template image, which is stored in a database. This template image is only defined once during the implementation process and is valid for all tests sharing the same format, independent of the number of strips and the drug configuration.

For defining the area corresponding to the strips in the actual processed image, a homography approach based on feature matching is used. For this purpose, the first step, which is done off-line, consists of calculating keypoints in the template image and extracting the corresponding descriptors. In this approach and with the aim of being computationally efficient, ORB (oriented FAST (Features from Accelerated Segment Test) and rotated BRIEF (Binary Robust Independent Elementary Features)) features are computed, and their descriptors are extracted. In [16], it is demonstrated how ORB is two orders of magnitude faster than SIFT. This is crucial in this kind of device for real-time processing.

ORB uses the well-known FAST keypoints [17,18] with a circular radius of nine (FAST-9) and introduces orientation to the keypoint by measuring the intensity centroid (oFAST). Then, the BRIEF descriptor [19] is computed, which consists of a bit string description of an image patch constructed from a set of binary intensity tests, using in this case a learning method for extracting subsets of uncorrelated binary tests (rBRIEF). The combination of oFAST keypoints and rBRIEF features conforms the final ORB descriptor and makes ORB features rotation invariant and robust to noise. Once the ORB keypoints and their descriptors have been extracted from the template image, they are stored in the smartphone.

**Figure 5:**
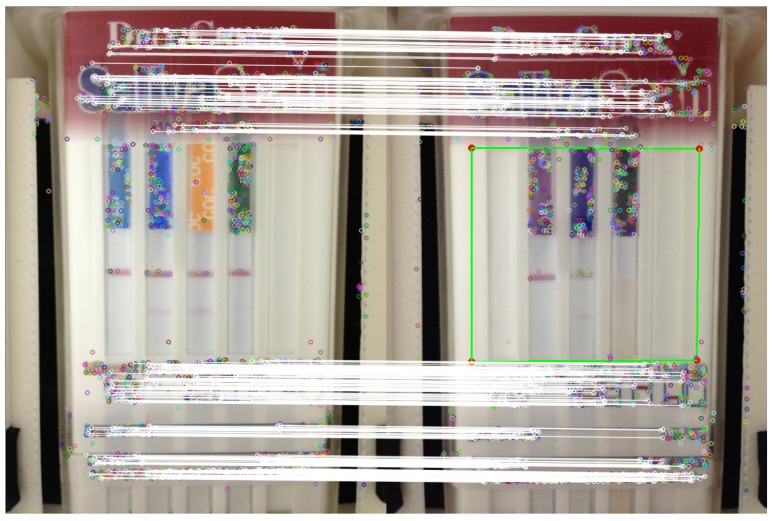
Matching process between oriented FAST and rotated BRIEF (ORB) descriptors in the template image (**left**) and original image (**right**). White lines denote the matched points. In green is depicted the searched region of interest that contains the strips in the actual processed image.

When a new image is captured from the device, the first step consists of extracting the ORB keypoints and their descriptors and matching them with the ones extracted from the template (Figure 5), which will provide the homography between both images, that is the transformation that converts a point in the template image to the corresponding point in the current image.

The matching process is divided into three steps:

- First, a brute force matching is computed. In this process, the descriptors are matched according to their Hamming distance, selecting for the next stage the ones that have the minimum Hamming [20] distance between them.
- Second, a mutual consistency step is done for removing those matches that do not correspond uniquely to their counterparts in the other image.
- Finally, with the point pairs from the previous step, a homography transformation is computed. In this step, a random sample consensus (RANSAC) [21] method is used for removing the ones that do not fit the rigid perspective transform, which are called outliers.

Once the homography between the template image and the current image has been computed, this transformation is applied to the selected four points in the template image that define the region of interest (ROI) of the strips (the area within the green border in Figure 5). This feature matching-based homography approach successfully deals with image noise, small input image displacements and rotations and different image resolutions.

The next stage in the strip segmentation process is to localize the colored area of each strip in the image. The drug strip configuration of the test is known in advance through a unique batch number provided by the test manufacturer, so it is only required to check that the number of detected strips in the image matches the test configuration and to localize these strips in the image. As the interpretation method for the tests containing an alcohol strip is specific to this drug, images containing an alcohol strip will be processed differently. According to this, the process for segmenting and localizing the colored area of each strip is as follows:

- If the image contains an alcohol strip, a thresholding procedure by the Otsu [22] method is applied on the R, G and B channels of the image ROI given by the homography. After that, two AND operations between Channels G and B, and G and R, are applied with the purpose of filtering the image.
- If the image does not contain an alcohol strip, the image is thresholded using the Otsu method in the R and B channels, and finally, an OR operation between these images is applied.

**Figure 6:**
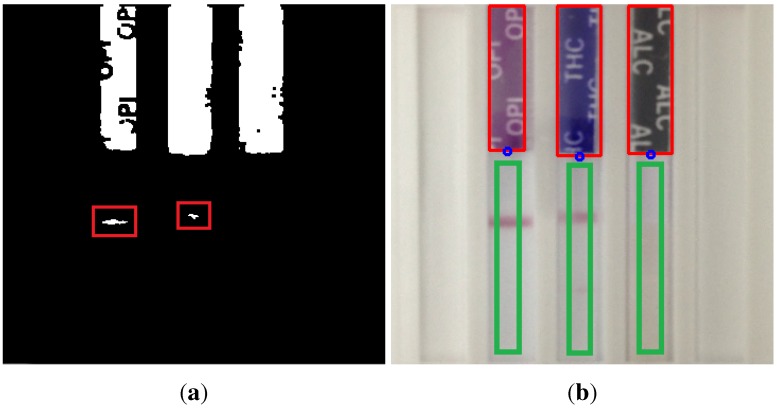
(**a**) Result of the segmentation process on a test image. Spurious contours, bounded in red, are filtered out during the post-processing stage; (**b**) Localization of the colored area of the strips after applying the segmentation process to a test image, bounded with a red rectangle. For each of these regions, its size is used together with the position of its bottom-middle point (depicted in blue) as parameters to automatically determine the region on which the lateral histogram will be computed, bounded with a green rectangle.

Finally, in both procedures, a morphological closing operation is applied to the resulting binary image of the previous steps. Then, in order to remove isolated pixels, a filter based on the number of pixels in each column is computed. For each of the columns, if the number of non-zero pixels is less than 10% of the total number of pixels in that column, the column is set to zero. An example of the result of the segmentation process on a test image is shown in Figure 6a.

The final step in the segmentation process consists of a post-processing stage, in which the contours of the previous binary image are computed. In this stage, several filters are applied to the computed contours:

- First, a position-based filter is applied. The purpose of this filter is to remove those contours whose centroid is located under the lower half of the computed ROI (area bounded in green in Figure 5) of the image. This is useful to filter out spurious contours, as the ones bounded in red in Figure 6a.
- Second, we apply a filter based on the area enclosed by each of the computed contours. Based on this, we remove those contours that satisfy: *A_i_* < 0.5 · *A_maxcontour_*, where *A_i_* is the area of the contour *i* and *A_maxcontour_* is the area of the largest contour. Again, the objective is to filter out small spurious contours, which do not correspond to the colored regions of each strip, which indicate the type of drug.

By applying the explained segmentation process, the colored region of each strip, which indicates the type of drug of each test strip, is finally localized within the ROI image, as shown in Figure 6b. For each of these extracted ROIs, its size is used together with the position of its bottom-middle point as parameters to automatically determine the region on which the lateral histogram will be computed, bounded in green in Figure 6b. By limiting the extraction of the lateral histogram to this area, the problems of pixel intensity variations due to the shadows of the edges of the strip is minimized.

### 4.3. Lateral Histogram Extraction and Preprocessing

The regions of interest that represent each segmented strip in the previous section need to be processed before the classification step takes place. Due to the different way in which alcohol and non-alcohol strips should be interpreted, both require different preprocessing stages.

In the case of a non-alcohol strip, the preprocessing stage consists of four steps described below, whose mission is to simplify the information given to the classifier as much as possible to increase its efficiency. All of these steps are done for each non-alcohol segmented strip.

The first step is the lateral histogram extraction from the area extracted during the segmentation stage. The lateral histogram is computed for each of these areas, where the average pixel intensity value for each image row (strip transversal direction) is computed according to:

(1) $$x_{HG}\left(i\right)=\frac{1}{n}\sum_{\forall j}s(i,j)$$

where *x_HG_*(*i*) is the lateral histogram extracted value in the *i* coordinate; *s*(*i*, *j*) is the (*i*, *j*) pixel intensity value; *i* are image rows (strip transversal direction); *j* are image columns (strip longitudinal direction); and *n* is the number of image columns inside the lateral histogram area.

Due to differences of image acquisition, raw lateral histograms do not usually have the same amount of bins. Therefore, an adjustment in the lateral histogram number of bins is set to an arbitrary number *n_bin_* = 100 through the use of quadratic interpolation. With this step, we ensure that the lateral histogram always has the same number of bins (set to *n_bin_* = 100), independent of the image used.

Then, to minimize the effect of the light intensity changes in the same segmented strip and between two different images, a RANSAC technique is applied. The proposed model to be fitted is a linear model *y* = *a* · *i* + *b*, where *y* is the lateral histogram value; *i* is the coordinate; and *a* and *b* are the estimated parameters. Then, the lateral histogram is recalculated:

(2) $$x_{HR}\left(i\right)=x_{HN}\left(i\right)-\left(a\cdot i+b\right)$$

where *x_HN_*(*i*) is the lateral histogram value in the *i* coordinate after the adjustment to *n_bin_* = 100.

As the lateral histogram peaks, which have the information of the test and control regions, are clearly separated, the fourth and last pre-processing step tries to exploit this information by lining up these peaks. The histogram *x_HR_* is divided into two similar parts. The first part uses bins from one to $\frac{n_{bin}}{2}\;=\;50$, while the last part uses bins from $\frac{n_{bin}}{2}=50$ to *n_bin_* = 100. The minima for each part, *h*_*min*1_(*i*) and *h*_*min*2_(*i*), respectively, are computed. The final lateral histogram *h_x_* is the conjunction of both minimum values with a range of 15 bins in each direction. That means the final lateral histogram *h_x_* has *n_bin−final_* = 62 bins. Note that the first 31 bins will correspond to the control line (C), and the remaining bins will correspond to the test line (T).

In the case of an alcohol strip, a different processing is done: the first and the second steps are analogous, but with *n_bin_* = 62. Then, a third step to reduce the light intensity differences between two different images is done. The average of only the first *n_av_* = 15 values of the lateral histogram is calculated and then subtracted from the original lateral histogram.

(3) $$h_{x}\left(i\right)=x_{HN}\left(i\right)-\frac{1}{n_{av}}Â\cdot \sum_{i=1}^{n_{av}}x_{HN}\left(i\right)$$

where *h_x_* is the lateral histogram value adjusted to *n_bin_* = 62 bins with no light intensity influence between two different images. This normalization is done only with respect to the first 15 values of the lateral histogram, because this part of the histogram has proven to account well for illumination changes along the strip. The normalization with respect to the mean of these 15 values helps to minimize the influence of differences in illumination along the strip.

### 4.4. Lateral Histogram Classification and Test Outcome

Three different supervised machine learning classifiers based on artificial neural networks (ANN) have been implemented for lateral histogram classification:

- A classifier for alcohol strip lateral histograms.
- A classifier for control lines in non-alcohol strip lateral histograms.
- A classifier for test lines in non-alcohol strip lateral histograms.

By dividing the classification task into three steps through the use of different classifiers, a better performance can be achieved due to its forced specialization. All three classifiers have the same kind of model, and only their structure (their size) and their parameters are different from one another.

Machine learning algorithms require a correct sample dataset in order to be trained. Supervised algorithms require examples of each of the desired classes to be learned in order to perform classification tasks.

The parameters of the classifier need to be set after the best structure is selected through a cross-validation algorithm. The available dataset is randomly, but uniformly divided into three sub-sets depending on its functionality. In our case, “training data” correspond to 70% of the available data and are used to adjust the parameters of the model; “validation data”, 15%, are used to check the model parameters, ensuring the correct training; finally, “test data”, 15%, test the performance of the classifier.

Five sequential algorithms have been considered for generating the classifiers. firstly, an unsupervised input data normalization is performed:

(4) $$x_{normalized}\left(i\right)=\frac{h_{x}\left(i\right)-{\bar{x}}_{train}\left(i\right)}{std(x_{train}\left(i\right))}$$

where *h_x_*(*i*) is the preprocessed lateral histogram value in the coordinate *i*; the subscript *train* indicates the “training data” set; ${\bar{x}}_{train}\left(i\right)$ stands for the mean value; and *std* stands for the standard deviation.

Secondly, an unsupervised data mapping is done:

(5) $$x_{mapped}\left(i\right)=2\cdot \frac{x_{normalized}\left(i\right)-min_{m,t}\left(i\right)}{max_{m,t}\left(i\right)-min_{m,t}\left(i\right)}-1$$

where *min_m,t_*(*i*) and *max_m,t_*(*i*) are the minimum and maximum values used for the mapping in the *i* coordinate and calculated with the “training data” set.

**Figure 7:**
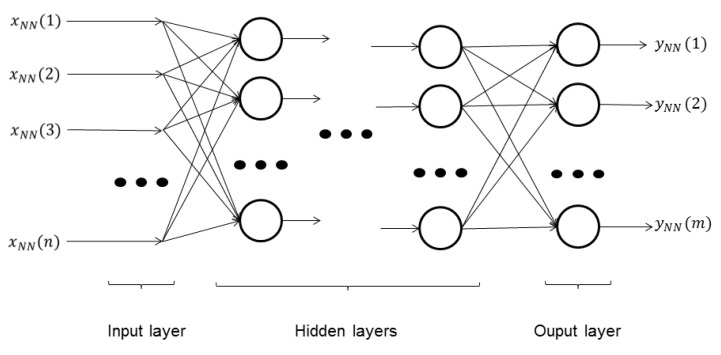
ANN-MLP structure diagram. Each neuron is represented by a circle, whose behavior is explained by Equation (6). The connections between the inputs, the neurons and the outputs are represented with arrows.

Then, a supervised ANN, multi-layer perceptron (MLP) [23], is first trained and afterwards executed to classify the data. This ANN is composed by several layers, called the “input layer”, “hidden layers” and the “output layer”, depicted in Figure 7. Each layer (except the “input layer”, which is only the input to the ANN) is formed by several neurons. Each neuron has a single output; multiple inputs (all of the outputs of the neurons of the previous layer); a weight value associated with each input; and a bias value. The behavior of each neuron, *i.e.*, how its output is computed based on its inputs and internal parameters, is given by:

(6) $$\text{output}=\text{tansig}\left(\sum_{\forall i}input\left(i\right)\cdot weight\left(i\right)+bias\right)$$

where tansig(*x*) is the hyperbolic tangent sigmoid function.

After the input ${\vec{x}}_{NN}={\vec{x}}_{mapped}$ is introduced to the ANN, it generates an output vector ${\vec{y}}_{NN}$. The number of neurons and its configuration (*i.e.*, the structure of the classifier) and the weights and biases of each neuron (*i.e.*, the parameters of the classifier) are calculated using the “training data” set.

In the next step, an unsupervised output data reverse mapping is done:

(7) $$y_{rev-mapped}\left(k\right)=\frac{y_{NN}\left(k\right)+1}{2({max}_{r,t}\left(k\right)-{min}_{r,t}\left(k\right))}+{min}_{r,t}\left(k\right)$$

where *max_r,t_*(*k*) and *min_r,t_*(*k*) are the maximum and minimum values used for reverse mapping in the *k* coordinate of the output and calculated with the “training data” set.

Finally, an unsupervised thresholding operation generates the classification output. The maximum of the reverse mapped output is calculated: $y_{candidate}=max\left({\vec{y}}_{rev-mapped}\right)$. Then, the thresholding operation is applied:

(8) $$y_{class}=\left\{\begin{array}{cc} k, & \text{if}\;y_{candidate}\geq y_{thres}\left(k\right)\\ \text{Undetermined}, & \text{otherwise} \end{array}\right.$$

This last step ensures that the algorithm gives only a trained output as a classification result if its confidence level is high enough, above a certain threshold. Otherwise, it will show a conservative behavior, outputting “undetermined” as the classification result.

#### 4.4.1. Classifier for Alcohol Strip Lateral Histograms

The input to this classifier is the 62-bin preprocessed lateral histogram for alcohol strips. The output of the trained classifier is a value that indicates the result of the test. The alcohol strips used in these experiments allow for the detection of five alcohol levels in saliva, depicted in Figure 2a. However, only three output categories have been considered: “positive (1)”, “negative (2)” or “undetermined (3)”. The reason for this simplification can be found in the market demand, where saliva-based tests compete with other diagnostic technologies. Breath-based analyzers are generally used for measuring blood alcohol content in massive testing, such as roadside tests, where a high accuracy is expected and expensive equipment can be used. However, low-cost saliva-based alcohol tests are used in situations where only a binary output is necessary, such as detoxification clinics.

**Figure 8:**
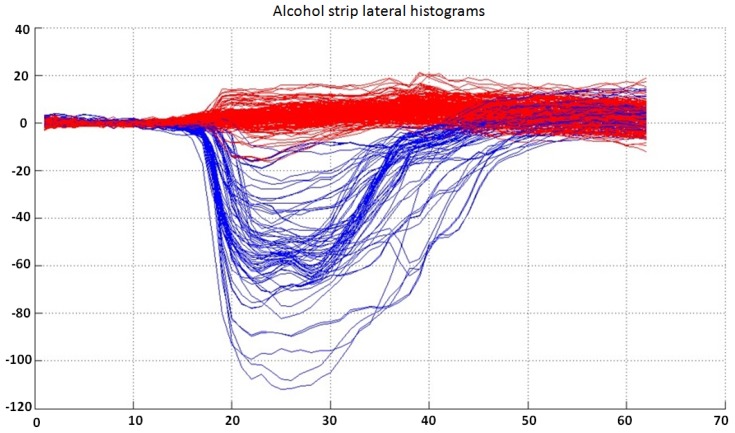
Lateral histograms used as the dataset for the classifier for the alcohol strip. Samples labeled as “negative” are represented in red, while “positive” samples are represented in blue. Note that the separability between classes is very good, and lateral histograms can be easily classified into three output categories (“positive”, “negative” or “undetermined”).

Due to the good separability between lateral histograms, these can be classified directly into the three output categories. The available complete dataset for this classifier is shown in Figure 8.

After an intensive training and output data analysis, it was determined that the best results were achieved using a classifier structure consisting of an ANN structure with 62 inputs, five neurons in a single hidden layer and two output neurons.

The evaluation of the classifier in the three data sub-sets after the structures and parameters are calculated is shown in Table 2. The performance of the classifier is excellent, showing no incorrect classifications.

**Table 2:** Evaluation of the classifier for alcohol strip lateral histograms. Note that confusion matrices have dimensions of 2 × 3, because “undetermined” samples were never considered as an input. “Undetermined” samples are just the classification output when a certain confidence level is not reached.

	Success	Confusion Matrix (P, N, U)
Training data (238 samples)	100%	$\left[\begin{array}{ccc} 52 & 0 & 0\\ 0 & 186 & 0 \end{array}\right]$
Validation data (50 samples)	100%	$\left[\begin{array}{ccc} 12 & 0 & 0\\ 0 & 38 & 0 \end{array}\right]$
Test data (50 samples)	100%	$\left[\begin{array}{ccc} 10 & 0 & 0\\ 0 & 40 & 0 \end{array}\right]$

#### 4.4.2. Classifier for Control Lines in Non-Alcohol Strip Lateral Histograms

**Figure 9:**
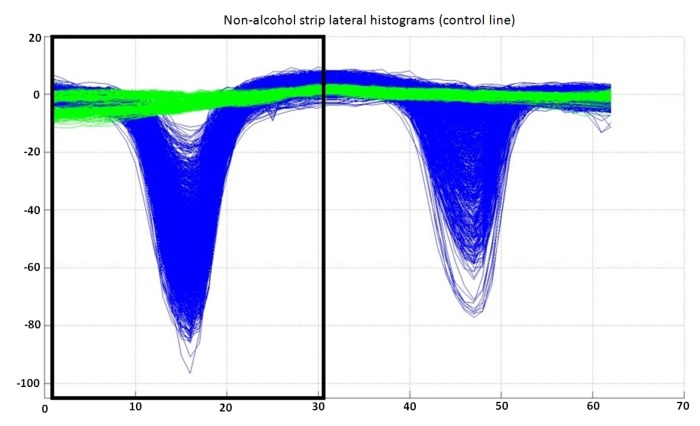
Lateral histograms used as the dataset for the classifier for control lines for non-alcohol strip lateral histograms. Samples labeled as “invalid” are represented in green, while “valid” samples are represented in blue. Note that only the first 31 bins of each lateral histogram are used by this classifier. It can also be observed that the separability between classes is very good, allowing the use of only three classes in this classifier (“valid”, “invalid” or “undetermined”).

The inputs to this classifier are the first 31 bins of the preprocessed lateral histogram of a non-alcohol strip (inside a black box in Figure 9). The output of the trained classifier can be “valid (1)”, “invalid (2)” or “undetermined (3)”. The complete dataset available for this classifier is shown in Figure 9.

After training and analyzing the output data, it was determined that the best results were achieved using a classifier structure consisting of an ANN structure with 31 inputs, a single neuron in a single hidden layer and two output neurons.

The evaluation of the classifier in the three data sub-sets after the structures and parameters are calculated is shown in Table 3. Again, the performance of the classifier is excellent, showing no incorrect classifications.

**Table 3:** Evaluation of the classifier for control lines in non-alcohol strip lateral histograms. Note that confusion matrices have dimensions of 2 × 3 because “undetermined” samples were never considered as an input. “Undetermined” samples are just the classification output when a certain confidence level is not reached.

	Success	Confusion Matrix (V, I, U)
Training data (1660 samples)	100%	$\left[\begin{array}{ccc} 1396 & 0 & 0\\ 0 & 264 & 0 \end{array}\right]$
Validation data (349 samples)	100%	$\left[\begin{array}{ccc} 293 & 0 & 0\\ 0 & 56 & 0 \end{array}\right]$
Test data (349 samples)	100%	$\left[\begin{array}{ccc} 293 & 0 & 0\\ 0 & 56 & 0 \end{array}\right]$

#### 4.4.3. Classifier for Test Lines in Non-Alcohol Strip Lateral Histograms

**Figure 10:**
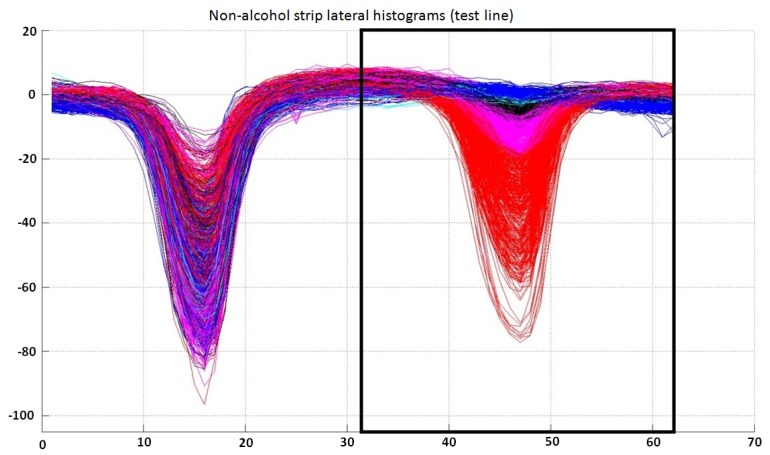
Lateral histogram samples used as training data for the classifier for test lines in non-alcohol strip lateral histograms. Samples labeled as “very negative” are represented in red; “negative” in magenta; “doubtful” in black; “positive” in cyan; and “very positive” in blue. Note that only the last 31 bins of each lateral histogram are used by this classifier. It can also be observed that the separability between classes is not very good, which justifies the use of six classes in this classifier (“very positive”, “positive”, “doubtful”, “negative”, “very negative” or “undetermined”).

**Figure 11:**
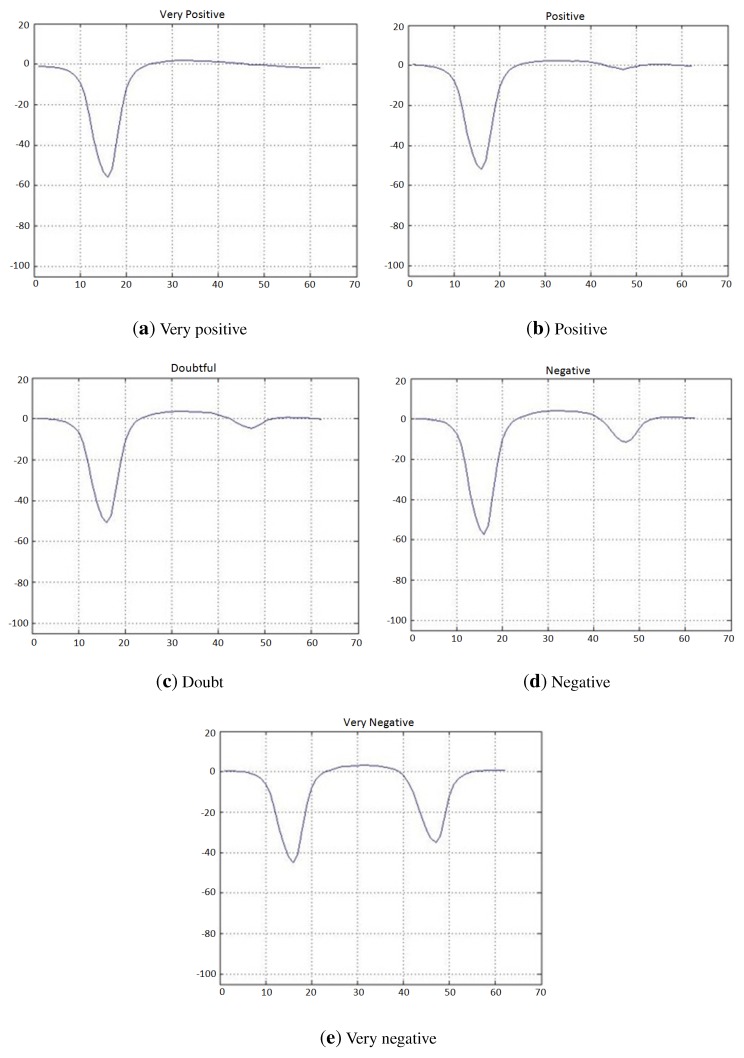
Lateral histograms representing a prototype of each labeled class used for the classifier for test lines in non-alcohol strips.

The inputs to this classifier are the last 31 bins of the preprocessed lateral histogram of a non-alcohol strip (inside a black box in Figure 10). The output of the trained classifier can fall into six different categories: “very positive (1)”, “positive (2)”, “doubtful (3)”, “negative (4)”, “very negative (5)” or “undetermined (6)”. Although there are only three possible output test results, “positive”, “negative” or “undetermined”, the internal use of a larger number of categories (see Figure 11) by the classifier allows one to have enhanced control of the treatment of doubtful cases, in order to adjust for a desired false positive or false negative ratio. The available complete dataset for this classifier is shown in Figure 10.

Once the training procedure was completed and the output data were analyzed, it was determined that the best results were achieved using a classifier structure consisting of an ANN structure with 31 inputs, two hidden layers with seven neurons each and five output neurons. The evaluation of the classifier in the three data sub-sets after the structures and parameters are calculated is shown in Table 4. The performance of the classifier is very good, showing only a few errors between adjacent labeled classes.

**Table 4:** Evaluation of the classifier for the test lines in non-alcohol strip lateral histograms. Note that confusion matrices have dimensions of 5 × 6 because “undetermined” samples were never considered as an input. “Undetermined” samples are just the classification output when a certain confidence level is not reached.

	Success	Confusion Matrix (VP, P, D, N, VN, U)
Training Data (1369 samples)	100%	$\left[\begin{array}{cccccc} 459 & 0 & 0 & 0 & 0 & 0\\ 0 & 122 & 0 & 0 & 0 & 0\\ 0 & 0 & 200 & 0 & 0 & 0\\ 0 & 0 & 0 & 258 & 0 & 0\\ 0 & 0 & 0 & 0 & 330 & 0 \end{array}\right]$
Validation data (293 samples)	91.809%	$\left[\begin{array}{cccccc} 94 & 2 & 0 & 0 & 0 & 0\\ 5 & 19 & 2 & 0 & 0 & 0\\ 0 & 4 & 39 & 4 & 0 & 0\\ 0 & 0 & 2 & 49 & 3 & 0\\ 0 & 0 & 0 & 2 & 68 & 0 \end{array}\right]$
Test data (293 samples)	89.761%	$\left[\begin{array}{cccccc} 93 & 2 & 0 & 0 & 0 & 0\\ 5 & 22 & 4 & 0 & 0 & 0\\ 0 & 1 & 35 & 8 & 0 & 0\\ 0 & 0 & 6 & 45 & 2 & 0\\ 0 & 0 & 0 & 2 & 68 & 0 \end{array}\right]$

## 5. System Evaluation Methodology

A study was conducted by an external company to verify the performance of the application with regards to the two following aspects:

- Verify the agreement between visual results obtained by human operators and those obtained by the test reader (agreement).
- Check the repeatability of the results interpreted by the test reader (precision).

The following values should be established as an outcome of the testing:

1. Provide the number of cases (%) in which the results obtained by the test reader agreed with the interpretation made by an operator by visual interpretation, on a given representative sample size (N) (at least two or three independent operators should be considered, to minimize bias or the impact of subjectivity).
2. Provide the number of cases (%) in which the test reader is able to repeat the same result (positive, negative or invalid) when interpreting any test, given a representative sample size (N).

Ninety SalivaScan double-sided tests were used (detection of six drugs + alcohol in oral fluid) with the following configuration: amphetamine, ketamine, cocaine and methamphetamine on one side and opiates, marijuana and alcohol on the other side, with the cutoff levels indicated in Table 1. Three detection levels were considered: negative, cutoff and 3× cutoff (saliva controls with three-times the cutoff concentration level).

In order to simulate the concentration on each detection level, positive and negative standard saliva controls were used. Such controls were sourced from Synergent Biochem Inc. ( Hawaiian Gardens, CA, USA) [24].

The visual and automated results obtained for the alcohol (ALC) strip were not recorded, as the interpretation for the results is made by comparison of the color changes in the reagent pad against a pattern and not by the interpretation of the control and test lines.

The results interpreted by the test reader were extracted using an iPhone 4 (partial test) and an iPhone 4S (full test), both running iOS Version 6.1.3.

### 5.1. Agreement Test

In the agreement test, the results of 30 tests per detection level were considered. Two human operators made the visual interpretation of the results obtained on each test, and the second operator obtained a result using the test reader in addition to his or her own interpretation. Each operator would log the results independently from each other. The detailed steps followed on each test were the following:

1. The reaction on each test was started after adding the corresponding positive or negative control depending on the cutoff level being tested.
2. Operator 1 waited 10 min for incubation of the result, interpreted it and logged the test outcome.
3. Operator 2 interpreted the visual result, independently from Operator 1, and logged the test outcome.
4. Operator 2 interpreted the result using the test reader and logged the result.

A retest was performed at the negative and 3× detection levels, using a different lot. On each level, 20 tests were performed.

### 5.2. Precision Test

Test devices from the agreement evaluation were randomly selected, three tests from each detection level (negative, cutoff, 3×). Test 1 corresponds to an (amphetamine (AMP), ketamine (KET), cocaine (COC), methamphetamine (MET)) configuration, while Tests 2 and 3 had an (opiates (OPI), THC, ALC) configuration. Each of these tests was processed 40 times repeatedly.

## 6. Results

### 6.1. Agreement Test Results

Agreement test results are summarized in Table 5. Results show that there are differences each time the visual interpretation is made by each human operator. Results also show how the disagreement between expert operators can be substantial (20% to 30%), which proves that interpreting the lines is not an easy task, especially in doubtful cases, and that specific training is necessary for correct interpretation. Usually, these interpretation differences occur when test lines are faint or remains of the reagent are present on the test strips. In such cases, it is normal that there are doubts about the result obtained, and this situation occurs normally when interpreting results at the cutoff level in contrast to the results obtained at the negative and 3× levels, which are expected to be easier to classify. It should be noted that the agreement on the THC strip in the levels of negative and 3× is very low, and not as expected (it should be near 100%), this was due to the fact that the results obtained on those levels showed a high number of faint test lines that caused doubts while the operators interpreted the results.

**Table 5:** Agreement test results summary. OP, operator. TR, test reader.

**Agreement OP1 *vs.* OP2 Test/Retest**	**AMP**	**KET**	**COC**	**MET**	**OPI**	**THC**	**Average**
Total Agreement (%)	93/100	93/100	100/100	89/95	100/80	87/100	94/96
Negative (%)	100/100	100/100	100/100	100/100	100/100	83/100	97/100
Cutoff (%)	80/-	90/-	100/-	67/-	100/-	97/-	89/-
3× (%)	100/100	90/100	100/100	100/90	100/60	80/100	95/92
**Agreement OP1 *vs.* TR Test/Retest**	**AMP**	**KET**	**COC**	**MET**	**OPI**	**THC**	**AVG**
Total Agreement (%)	87/100	92/100	100/95	91/100	98/95	94/98	93/98
Negative (%)	100/100	100/100	100/100	100/100	100/100	100/100	100/100
Cutoff (%)	60/-	93/-	100/-	73/-	93/-	85/-	84/-
3× (%)	100/100	83/100	100/90	100/100	100/90	96/95	97/96
**Agreement OP2 *vs.* TR Test/Retest**	**AMP**	**KET**	**COC**	**MET**	**OPI**	**THC**	**AVG**
Total Agreement (%)	87/100	92/100	100/95	89/95	98/75	69/98	89/94
Negative (%)	100/100	100/100	100/100	100/100	100/100	71/100	95/100
Cutoff (%)	60/-	97/-	100/-	67/-	93/-	60/-	79/-
3× (%)	100/100	79/100	100/90	100/90	100/50	76/95	93/88
**OP1 and OP2 *vs.* TR Test/Retest**	**AMP**	**KET**	**COC**	**MET**	**OPI**	**THC**	**AVG**
Total Agreement (%)	90/100	96/100	100/95	96/100	96/95	72/98	91/98
Negative (%)	100/100	100/100	100/100	100/100	93/100	70/100	94/100
Cutoff (%)	70/-	100/-	100/-	87/-	93/-	60/-	85/-
3× (%)	100/100	86/100	100/90	100/100	100/90	86/95	95/96

There was a large number of results obtained in the rapid test in which the test reader (TR) judged the result as negative, while the operators (OPs) judged such results as positives (OPs positive–TR negative). This was caused by the fact that the test lines in some strips were very faint, which induced the operators to judge the results incorrectly as positive when these should have been labeled as negative, as determined by the test reader.

The cases when the operators judged the result as negative and the test reader interpreted it as positive can be explained by the development of faint test lines.

There are discrepancies in the agreement between operators (OP1 *vs.* OP2) when interpreting the results on the OPI strip, especially at the 3× detection level; this may be due to the appearance of stains or color remaining on the reagent strip.

The agreement of Operator 2 is substantially lower than that of Operator 1. It can be seen that Operator 2 had some doubts while interpreting the results for the OPI strip at the level of 3×.

As can be seen, all errors correspond to the strips tested where it was expected to obtain positive results (3×). At such levels, the test reader interpreted the result as positive, while the operators judged the result as negative. This discrepancy may probably be associated with the presence of color remaining on the strip, which might give the impression to the operators of a test line, while such lines did not have the typical characteristics to be considered as test lines.

The cases in which both operators did not agree on the results obtained by the test reader can be classified in the categories shown in Table 6, as well as the results as a percentage of the total number of strips evaluated.

**Table 6:** Disagreement and adjusted agreement test results summary.

Both Operators *vs.* Test Reader	AMP	KET	COC	MET	OPI	THC	Total
Disagreements OPs negative–TR positive count (%)/retest count (%)	0 (0)/0 (0)	0 (0)/0 (0)	0 (0)/2 (5)	0 (0)/0 (0)	0 (0)/2 (5)	3 (3)/1 (3)	3 (1)/5 (2)
Disagreements OPs positive–TR negative count (%)/retest count (%)	9 (10)/0 (0)	4 (4)/0 (0)	0 (0)/0 (0)	4 (4)/0 (0)	2 (2)/0 (0)	20 (22)/0 (0)	39 (7)/0 (0)
Disagreements TR error count (%)/retest count (%)	0 (0)/0 (0)	0 (0)/0 (0)	0 (0)/0 (0)	0 (0)/0 (0)	2 (2)/0 (0)	2 (2)/0 (0)	4 (1)/0 (0)
Total disagreements count/retest count	9/0	4/0	0/2	4/0	4/2	25/1	46/5
Total adjusted agreement (%)/retest (%)	100/100	100/100	100/95	100/100	98/95	94/98	99/98
Total test count/retest count	89/40	89/40	89/40	89/40	89/40	89/40	534/240

Excluding the cases in which the operators indicated positive when the test reader indicated negative and counting them as interpretation errors attributable to the operators, the total adjusted agreement (excluding cases when OPs positive and TR negative) has been computed and is shown in Table 6.

### 6.2. Precision Test Results

The results indicated in Table 7 were obtained once the tests were processed repeatedly using the test reader for each detection level. The cases in which the test reader interpreted the result as “negative” when the operator interpreted it as “positive” correspond to strips showing very faint test lines, especially at “cutoff Test 1” and “3× Test 1” on the strips KET and OPI.

**Table 7:** Precision test results.

Detection Level	No. Tests	No. Strips per Side	Total Strips ^1^	No. of Times That TR Gave The Same Result for a Given Strip ^2^	Precision (%)
Negative test 1/2/3	40/40/40	4/2/2	156/78/78	156/78/78	100/100/100
Precision for negative			312	312	100
Cutoff test 1/2/3	40/40/40	4/2/2	160/80/80	149/78/71	93/98/89
Precision for cutoff			320	298	94
3× test 1/2/3	40/40/40	4/2/2	156/80/80	137/80/80	88/100/100
Precision for 3×			316	297	94
Total	360		948	907	96

^1^ Total number of strips correctly interpreted; invalid strips or errors excluded; ^2^ in the case of different results, the maximum value was taken.

## 7. Conclusions

An innovative, low-cost, portable approach for the rapid interpretation of lateral flow saliva test results in drug-of-abuse detection based on the use of commonly-available smartphone devices has been presented and evaluated. A small inexpensive light box is used to control image quality parameters during the acquisition. This solution reuses an existing smartphone, and there is no additional equipment needed, besides the light box, which costs a fraction of the price for similar products on the market, with prices ranging around 3000 EUR/device. In order to segment the strips, images are first pre-processed to correct for small displacements and/or rotations with an ORB feature-based matching and homography strategy, and the strips corresponding to the different substances to be detected are segmented using color features and morphological operations. Finally, a lateral histogram containing the saliva test lines’ intensity profile is extracted. Lateral histograms are then classified with a machine learning-based procedure, including unsupervised data normalization and classification using a multilayer perceptron artificial neural network (MLP-ANN). The implemented solution can be adapted to any line-based or color-based lateral flow test on the market. System agreement and precision tests were run for system evaluation, showing great agreement between the visual results obtained by human operators and those obtained by the test reader app, while showing high repeatability. The objective of the work is to demonstrate that the test reader is able to obtain the same result (or better) than a trained operator, therefore reducing the subjectivity of the analysis by standardizing the test interpretation conditions (illumination, test to image sensor distance, *etc.*) and by using a deterministic algorithm to obtain the results. In this sense, any operator independent of his/her level of experience can rely on the results obtained by the test reader. The system is automatic, allowing one to systematize the collection and analysis of the data in real time, removing the risks of manual results’ management and allowing for centralized processing. The mentioned features can be very useful in places where there is no qualified staff and rapid detection is needed, such as on-site detection performed by a police officer or a first-aid operator.
